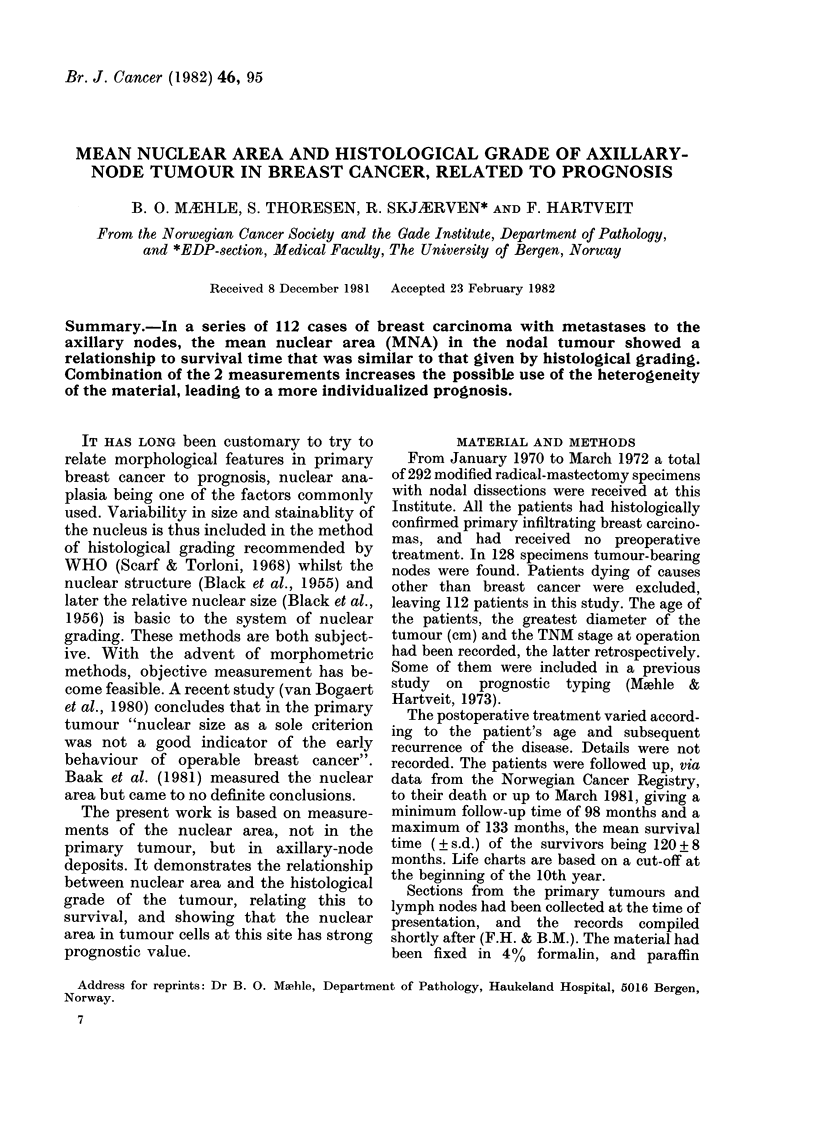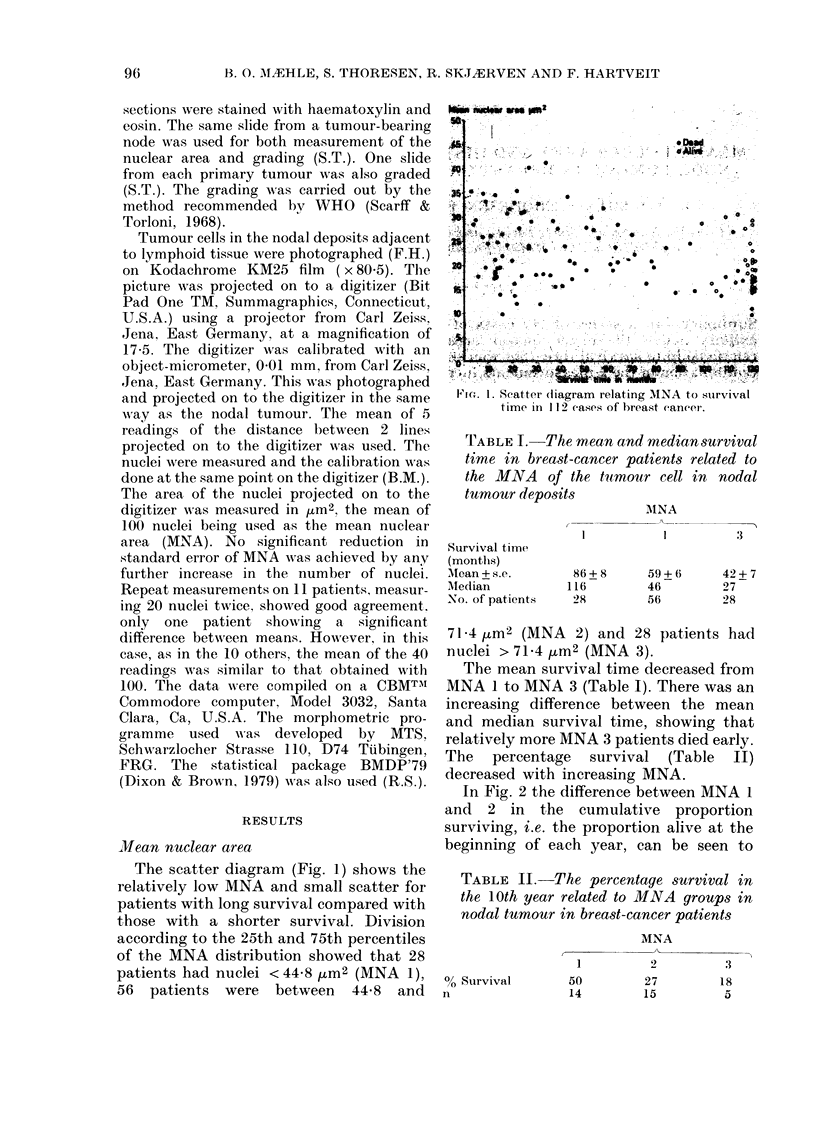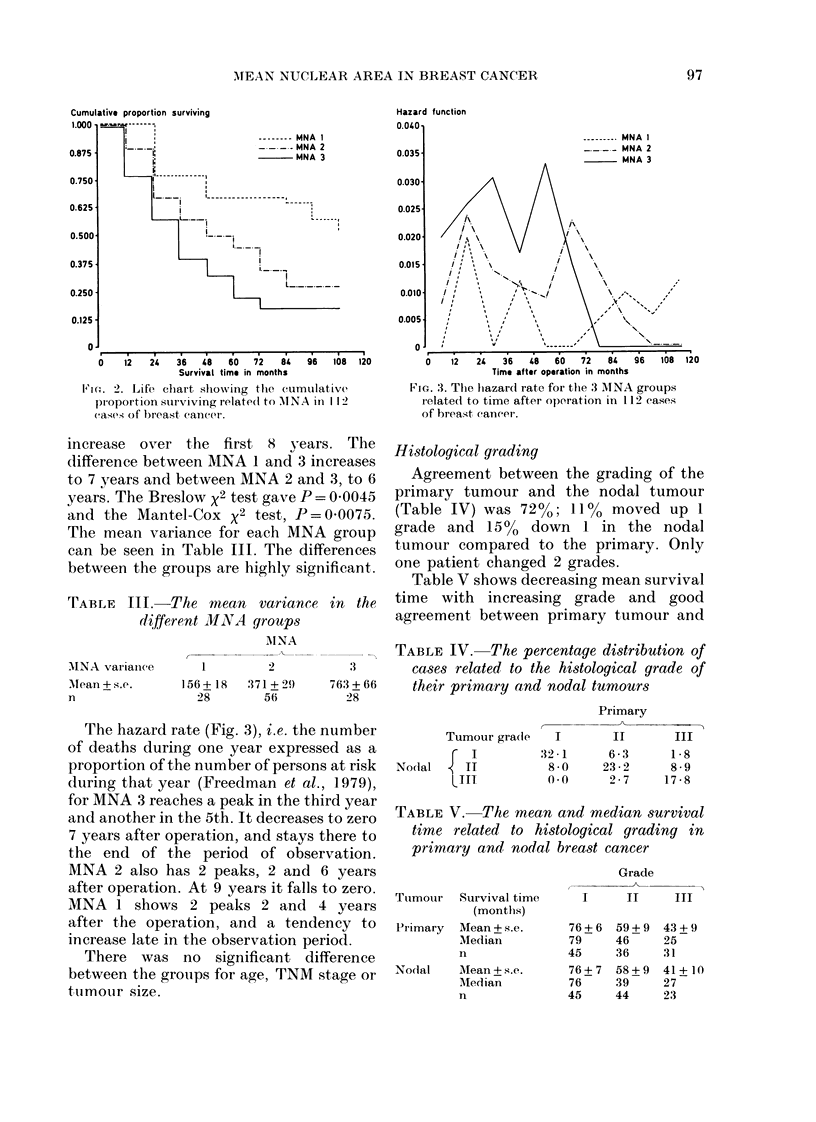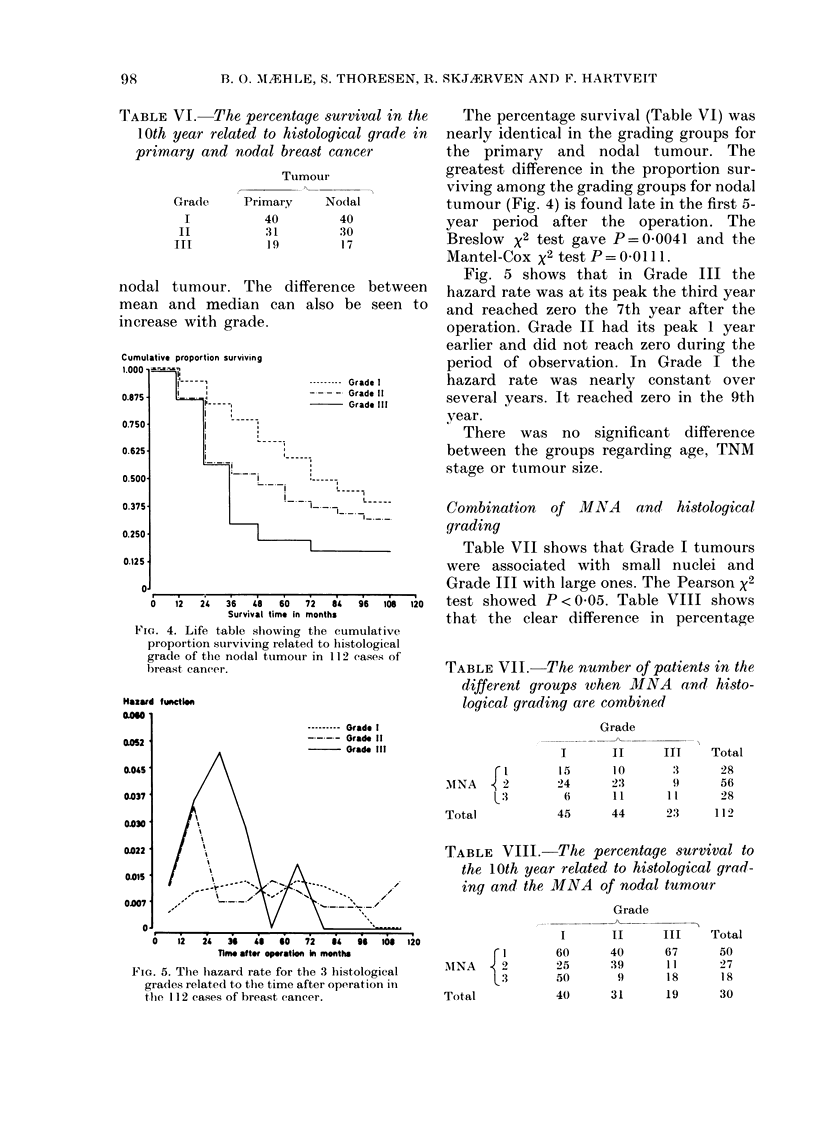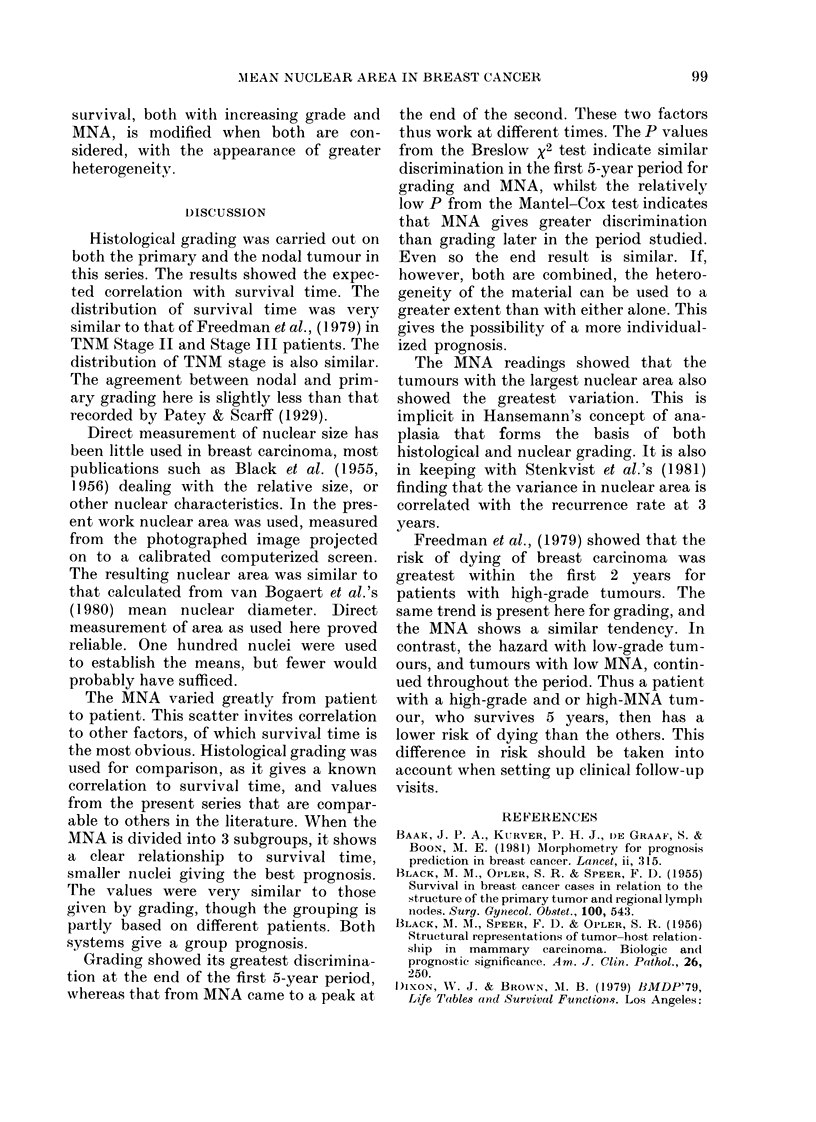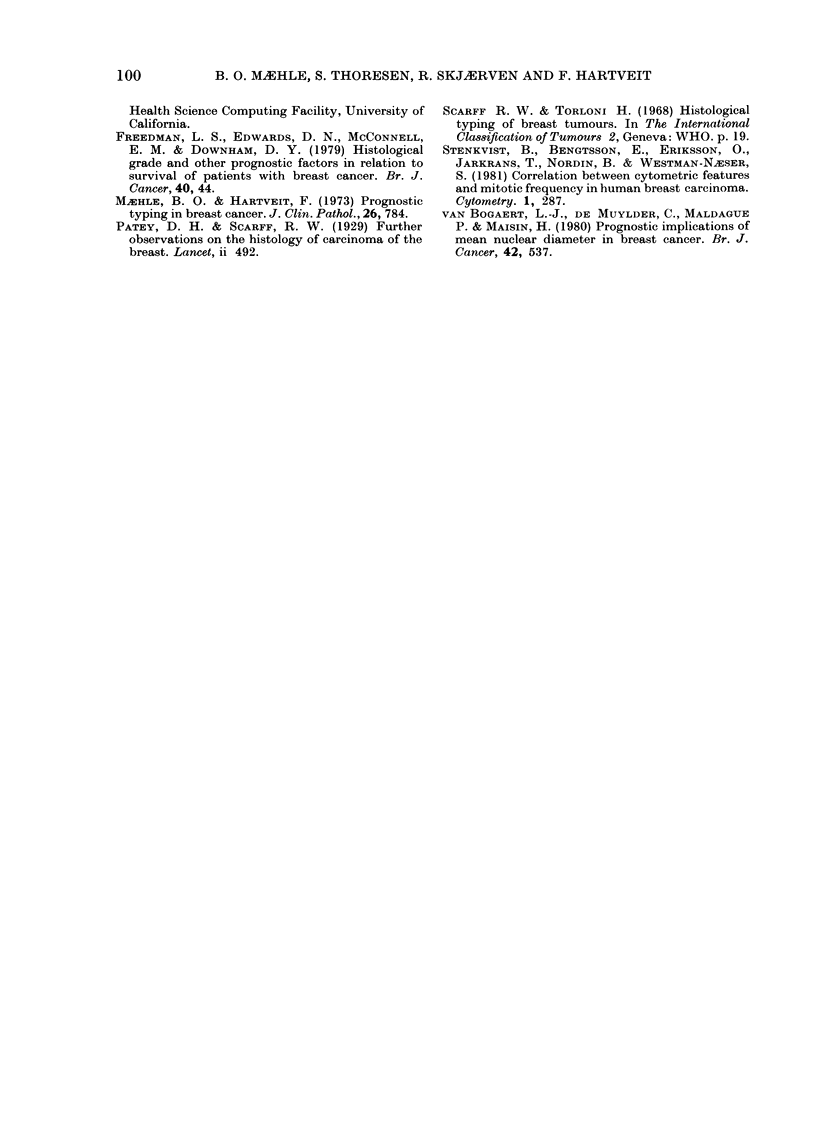# Mean nuclear area and histological grade of axillary-node tumour in breast cancer, related to prognosis

**DOI:** 10.1038/bjc.1982.170

**Published:** 1982-07

**Authors:** B. O. Mæhle, S. Thoresen, R. Skjærven, F. Hartveit

## Abstract

In a series of 112 cases of breast carcinoma with metastases to the axillary nodes, the mean nuclear area (MNA) in the nodal tumour showed a relationship to survival time that was similar to that given by histological grading. Combination of the 2 measurements increases the possible use of the heterogeneity of the material, leading to a more individualized prognosis.


*Br. J. Cancer* (1982) **46**, 95

### MEAN NUCLEAR AREA AND HISTOLOGICAL GRADE OF AXILLARY-NODE TUMOUR IN BREAST CANCER, RELATED TO PROGNOSIS

B. O. MÆHLE, S. THORESEN, R. SKJÆRVEN* AND F. HARTVEIT

*From the Norwegian Cancer Society and the Gade Institute, Department of Pathology, and *EDP-section, Medical Faculty, The University of Bergen, Norway*

Received 8 December 1981 Accepted 23 February 1982

**Summary.—In a series of 112 cases of breast carcinoma with metastases to the axillary nodes, the mean nuclear area (MNA) in the nodal tumour showed a relationship to survival time that was similar to that given by histological grading. Combination of the 2 measurements increases the possible use of the heterogeneity of the material, leading to a more individualized prognosis.**

IT HAS LONG been customary to try to relate morphological features in primary breast cancer to prognosis, nuclear anaplasia being one of the factors commonly used. Variability in size and stainablity of the nucleus is thus included in the method of histological grading recommended by WHO (Scarf & Torloni, 1968) whilst the nuclear structure (Black *et al.*, 1955) and later the relative nuclear size (Black *et al.*, 1956) is basic to the system of nuclear grading. These methods are both subjective. With the advent of morphometric methods, objective measurement has become feasible. A recent study (van Bogaert *et al.*, 1980) concludes that in the primary tumour "nuclear size as a sole criterion was not a good indicator of the early behaviour of operable breast cancer". Baak *et al.* (1981) measured the nuclear area but came to no definite conclusions.

The present work is based on measurements of the nuclear area, not in the primary tumour, but in axillary-node deposits. It demonstrates the relationship between nuclear area and the histological grade of the tumour, relating this to survival, and showing that the nuclear area in tumour cells at this site has strong prognostic value.

### MATERIAL AND METHODS

From January 1970 to March 1972 a total of 292 modified radical-mastectomy specimens with nodal dissections were received at this Institute. All the patients had histologically confirmed primary infiltrating breast carcinomas, and had received no preoperative treatment. In 128 specimens tumour-bearing nodes were found. Patients dying of causes other than breast cancer were excluded, leaving 112 patients in this study. The age of the patients, the greatest diameter of the tumour (cm) and the TNM stage at operation had been recorded, the latter retrospectively. Some of them were included in a previous study on prognostic typing (Mæhle & Hartveit, 1973).

The postoperative treatment varied according to the patient's age and subsequent recurrence of the disease. Details were not recorded. The patients were followed up, *via* data from the Norwegian Cancer Registry, to their death or up to March 1981, giving a minimum follow-up time of 98 months and a maximum of 133 months, the mean survival time ($\pm$s.d.) of the survivors being $120 \pm 8$ months. Life charts are based on a cut-off at the beginning of the 10th year.

Sections from the primary tumours and lymph nodes had been collected at the time of presentation, and the records compiled shortly after (F.H. & B.M.). The material had been fixed in 4% formalin, and paraffin

Address for reprints: Dr B. O. Mæhle, Department of Pathology, Haukeland Hospital, 5016 Bergen, Norway.

sections were stained with haematoxylin and eosin. The same slide from a tumour-bearing node was used for both measurement of the nuclear area and grading (S.T.). One slide from each primary tumour was also graded (S.T.). The grading was carried out by the method recommended by WHO (Scarff & Torloni, 1968).

Tumour cells in the nodal deposits adjacent to lymphoid tissue were photographed (F.H.) on Kodachrome KM25 film (×80·5). The picture was projected on to a digitizer (Bit Pad One TM, Summagraphics, Connecticut, U.S.A.) using a projector from Carl Zeiss, Jena, East Germany, at a magnification of 17·5. The digitizer was calibrated with an object-micrometer, 0·01 mm, from Carl Zeiss, Jena, East Germany. This was photographed and projected on to the digitizer in the same way as the nodal tumour. The mean of 5 readings of the distance between 2 lines projected on to the digitizer was used. The nuclei were measured and the calibration was done at the same point on the digitizer (B.M.). The area of the nuclei projected on to the digitizer was measured in $\mu m^2$, the mean of 100 nuclei being used as the mean nuclear area (MNA). No significant reduction in standard error of MNA was achieved by any further increase in the number of nuclei. Repeat measurements on 11 patients, measuring 20 nuclei twice, showed good agreement, only one patient showing a significant difference between means. However, in this case, as in the 10 others, the mean of the 40 readings was similar to that obtained with 100. The data were compiled on a CBM™ Commodore computer, Model 3032, Santa Clara, Ca, U.S.A. The morphometric programme used was developed by MTS, Schwarzlocher Strasse 110, D74 Tübingen, FRG. The statistical package BMDP'79 (Dixon & Brown, 1979) was also used (R.S.).

### RESULTS

### *Mean nuclear area*

The scatter diagram (Fig. 1) shows the relatively low MNA and small scatter for patients with long survival compared with those with a shorter survival. Division according to the 25th and 75th percentiles of the MNA distribution showed that 28 patients had nuclei $< 44·8\ \mu m^2$ (MNA 1), 56 patients were between 44·8 and 71·4 $\mu m^2$ (MNA 2) and 28 patients had nuclei $> 71·4\ \mu m^2$ (MNA 3).

FIG. 1. Scatter diagram relating MNA to survival time in 112 cases of breast cancer.

TABLE I.—*The mean and median survival time in breast-cancer patients related to the MNA of the tumour cell in nodal tumour deposits*

| | MNA | | |
|---|---|---|---|
| | 1 | 1 | 3 |
| Survival time (months) | | | |
| Mean ± s.e. | 86 ± 8 | 59 ± 6 | 42 ± 7 |
| Median | 116 | 46 | 27 |
| No. of patients | 28 | 56 | 28 |

The mean survival time decreased from MNA 1 to MNA 3 (Table I). There was an increasing difference between the mean and median survival time, showing that relatively more MNA 3 patients died early. The percentage survival (Table II) decreased with increasing MNA.

In Fig. 2 the difference between MNA 1 and 2 in the cumulative proportion surviving, *i.e.* the proportion alive at the beginning of each year, can be seen to

TABLE II.—*The percentage survival in the 10th year related to MNA groups in nodal tumour in breast-cancer patients*

| | MNA | | |
|---|---|---|---|
| | 1 | 2 | 3 |
| % Survival | 50 | 27 | 18 |
| n | 14 | 15 | 5 |

FIG. 2. Life chart showing the cumulative proportion surviving related to MNA in 112 cases of breast cancer.

FIG. 3. The hazard rate for the 3 MNA groups related to time after operation in 112 cases of breast cancer.

increase over the first 8 years. The difference between MNA 1 and 3 increases to 7 years and between MNA 2 and 3, to 6 years. The Breslow $\chi^2$ test gave $P = 0{\cdot}0045$ and the Mantel-Cox $\chi^2$ test, $P = 0{\cdot}0075$. The mean variance for each MNA group can be seen in Table III. The differences between the groups are highly significant.

TABLE III.—*The mean variance in the different MNA groups*

| | MNA | | |
|---|---|---|---|
| MNA variance | 1 | 2 | 3 |
| Mean ± s.e. | 156 ± 18 | 371 ± 29 | 763 ± 66 |
| n | 28 | 56 | 28 |

The hazard rate (Fig. 3), *i.e.* the number of deaths during one year expressed as a proportion of the number of persons at risk during that year (Freedman *et al.*, 1979), for MNA 3 reaches a peak in the third year and another in the 5th. It decreases to zero 7 years after operation, and stays there to the end of the period of observation. MNA 2 also has 2 peaks, 2 and 6 years after operation. At 9 years it falls to zero. MNA 1 shows 2 peaks 2 and 4 years after the operation, and a tendency to increase late in the observation period.

There was no significant difference between the groups for age, TNM stage or tumour size.

*Histological grading*

Agreement between the grading of the primary tumour and the nodal tumour (Table IV) was 72%; 11% moved up 1 grade and 15% down 1 in the nodal tumour compared to the primary. Only one patient changed 2 grades.

Table V shows decreasing mean survival time with increasing grade and good agreement between primary tumour and

TABLE IV.—*The percentage distribution of cases related to the histological grade of their primary and nodal tumours*

| | | Primary | | |
|---|---|---|---|---|
| | Tumour grade | I | II | III |
| Nodal | I | 32·1 | 6·3 | 1·8 |
| | II | 8·0 | 23·2 | 8·9 |
| | III | 0·0 | 2·7 | 17·8 |

TABLE V.—*The mean and median survival time related to histological grading in primary and nodal breast cancer*

| | | Grade | | |
|---|---|---|---|---|
| Tumour | Survival time (months) | I | II | III |
| Primary | Mean ± s.e. | 76 ± 6 | 59 ± 9 | 43 ± 9 |
| | Median | 79 | 46 | 25 |
| | n | 45 | 36 | 31 |
| Nodal | Mean ± s.e. | 76 ± 7 | 58 ± 9 | 41 ± 10 |
| | Median | 76 | 39 | 27 |
| | n | 45 | 44 | 23 |

TABLE VI.—*The percentage survival in the 10th year related to histological grade in primary and nodal breast cancer*

| | Tumour | |
|---|---|---|
| Grade | Primary | Nodal |
| I | 40 | 40 |
| II | 31 | 30 |
| III | 19 | 17 |

nodal tumour. The difference between mean and median can also be seen to increase with grade.

FIG. 4. Life table showing the cumulative proportion surviving related to histological grade of the nodal tumour in 112 cases of breast cancer.

FIG. 5. The hazard rate for the 3 histological grades related to the time after operation in the 112 cases of breast cancer.

The percentage survival (Table VI) was nearly identical in the grading groups for the primary and nodal tumour. The greatest difference in the proportion surviving among the grading groups for nodal tumour (Fig. 4) is found late in the first 5-year period after the operation. The Breslow $\chi^2$ test gave $P = 0{\cdot}0041$ and the Mantel-Cox $\chi^2$ test $P = 0{\cdot}0111$.

Fig. 5 shows that in Grade III the hazard rate was at its peak the third year and reached zero the 7th year after the operation. Grade II had its peak 1 year earlier and did not reach zero during the period of observation. In Grade I the hazard rate was nearly constant over several years. It reached zero in the 9th year.

There was no significant difference between the groups regarding age, TNM stage or tumour size.

### *Combination of MNA and histological grading*

Table VII shows that Grade I tumours were associated with small nuclei and Grade III with large ones. The Pearson $\chi^2$ test showed $P < 0{\cdot}05$. Table VIII shows that the clear difference in percentage

TABLE VII.—*The number of patients in the different groups when MNA and histological grading are combined*

| | | Grade | | | |
|---|---|---|---|---|---|
| | | I | II | III | Total |
| MNA | 1 | 15 | 10 | 3 | 28 |
| | 2 | 24 | 23 | 9 | 56 |
| | 3 | 6 | 11 | 11 | 28 |
| Total | | 45 | 44 | 23 | 112 |

TABLE VIII.—*The percentage survival to the 10th year related to histological grading and the MNA of nodal tumour*

| | | Grade | | | |
|---|---|---|---|---|---|
| | | I | II | III | Total |
| MNA | 1 | 60 | 40 | 67 | 50 |
| | 2 | 25 | 39 | 11 | 27 |
| | 3 | 50 | 9 | 18 | 18 |
| Total | | 40 | 31 | 19 | 30 |

survival, both with increasing grade and MNA, is modified when both are considered, with the appearance of greater heterogeneity.

### DISCUSSION

Histological grading was carried out on both the primary and the nodal tumour in this series. The results showed the expected correlation with survival time. The distribution of survival time was very similar to that of Freedman *et al.*, (1979) in TNM Stage II and Stage III patients. The distribution of TNM stage is also similar. The agreement between nodal and primary grading here is slightly less than that recorded by Patey & Scarff (1929).

Direct measurement of nuclear size has been little used in breast carcinoma, most publications such as Black *et al.* (1955, 1956) dealing with the relative size, or other nuclear characteristics. In the present work nuclear area was used, measured from the photographed image projected on to a calibrated computerized screen. The resulting nuclear area was similar to that calculated from van Bogaert *et al.*'s (1980) mean nuclear diameter. Direct measurement of area as used here proved reliable. One hundred nuclei were used to establish the means, but fewer would probably have sufficed.

The MNA varied greatly from patient to patient. This scatter invites correlation to other factors, of which survival time is the most obvious. Histological grading was used for comparison, as it gives a known correlation to survival time, and values from the present series that are comparable to others in the literature. When the MNA is divided into 3 subgroups, it shows a clear relationship to survival time, smaller nuclei giving the best prognosis. The values were very similar to those given by grading, though the grouping is partly based on different patients. Both systems give a group prognosis.

Grading showed its greatest discrimination at the end of the first 5-year period, whereas that from MNA came to a peak at the end of the second. These two factors thus work at different times. The $P$ values from the Breslow $\chi^2$ test indicate similar discrimination in the first 5-year period for grading and MNA, whilst the relatively low $P$ from the Mantel–Cox test indicates that MNA gives greater discrimination than grading later in the period studied. Even so the end result is similar. If, however, both are combined, the heterogeneity of the material can be used to a greater extent than with either alone. This gives the possibility of a more individualized prognosis.

The MNA readings showed that the tumours with the largest nuclear area also showed the greatest variation. This is implicit in Hansemann's concept of anaplasia that forms the basis of both histological and nuclear grading. It is also in keeping with Stenkvist *et al.*'s (1981) finding that the variance in nuclear area is correlated with the recurrence rate at 3 years.

Freedman *et al.*, (1979) showed that the risk of dying of breast carcinoma was greatest within the first 2 years for patients with high-grade tumours. The same trend is present here for grading, and the MNA shows a similar tendency. In contrast, the hazard with low-grade tumours, and tumours with low MNA, continued throughout the period. Thus a patient with a high-grade and or high-MNA tumour, who survives 5 years, then has a lower risk of dying than the others. This difference in risk should be taken into account when setting up clinical follow-up visits.

### REFERENCES

Baak, J. P. A., Kurver, P. H. J., de Graaf, S. & Boon, M. E. (1981) Morphometry for prognosis prediction in breast cancer. *Lancet*, ii, 315.

Black, M. M., Opler, S. R. & Speer, F. D. (1955) Survival in breast cancer cases in relation to the structure of the primary tumor and regional lymph nodes. *Surg. Gynecol. Obstet.*, **100**, 543.

Black, M. M., Speer, F. D. & Opler, S. R. (1956) Structural representations of tumor–host relationship in mammary carcinoma. Biologic and prognostic significance. *Am. J. Clin. Pathol.*, **26**, 250.

Dixon, W. J. & Brown, M. B. (1979) *BMDP'79, Life Tables and Survival Functions*. Los Angeles:

Health Science Computing Facility, University of California.
FREEDMAN, L. S., EDWARDS, D. N., MCCONNELL, E. M. & DOWNHAM, D. Y. (1979) Histological grade and other prognostic factors in relation to survival of patients with breast cancer. *Br. J. Cancer*, **40**, 44.
MÆHLE, B. O. & HARTVEIT, F. (1973) Prognostic typing in breast cancer. *J. Clin. Pathol.*, **26**, 784.
PATEY, D. H. & SCARFF, R. W. (1929) Further observations on the histology of carcinoma of the breast. *Lancet*, ii 492.
SCARFF R. W. & TORLONI H. (1968) Histological typing of breast tumours. In *The International Classification of Tumours 2*, Geneva: WHO. p. 19.
STENKVIST, B., BENGTSSON, E., ERIKSSON, O., JARKRANS, T., NORDIN, B. & WESTMAN-NÆSER, S. (1981) Correlation between cytometric features and mitotic frequency in human breast carcinoma. *Cytometry*. **1**, 287.
VAN BOGAERT, L.-J., DE MUYLDER, C., MALDAGUE P. & MAISIN, H. (1980) Prognostic implications of mean nuclear diameter in breast cancer. *Br. J. Cancer*, **42**, 537.